# Surgical management of catastrophic caustic ingestion in acute phase: A case report and review of the literature

**DOI:** 10.1016/j.ijscr.2024.110188

**Published:** 2024-08-17

**Authors:** Ramin Bozorgmehr, Ahmadreza Sadeghi, Mohammad Sajad Bagheri Chokami, Mohammad Pourhooshmandi, Javad Zebarjadi Bagherpour, Zahra Iravani

**Affiliations:** a Department of General Surgery, Madani Hospital, Alborz University of Medical Sciences, Karaj, Iran; b Student Research Committee, Alborz University of Medical Sciences, Karaj, Iran

**Keywords:** Case report, Caustic, Corrosive, Ingestion, Injury, Pancreaticoduodenectomy

## Abstract

**Introduction:**

Caustic ingestion almost occurs accidentally in children and mostly intentionally in adults. The ingestion of caustic substances can cause various degrees of damage to the gastrointestinal tract. Depending on the severity of the injury, surgery may be a part of the treatment plan.

**Presentation of case:**

A 32-year-old man was referred to our hospital after swallowing drain cleaner. Due to evidence of peritonitis and endoscopy results, he underwent emergency surgery. During the surgery, necrotic parts, including the esophagus, stomach, duodenum, head of the pancreas, and initial part of the jejunum, were resected. Then, after six months, colon interposition surgery was done to reconstruct the gastrointestinal tract.

**Discussion:**

Like trauma patients, managing patients with caustic injuries begins with an initial survey of the airway, breathing, and circulation status. In the first 48 h, early esophagoscopy is indicated to evaluate the amount of injury. Evidence of transmural necrosis or perforation is the most important indication for surgery, and surgical procedures are specific to each patient. Esophagogastrectomy is the most common surgery in cases of severe gastrointestinal injuries, but removing more abdominal organs may be needed in fewer cases.

**Conclusion:**

This case report underscores the urgent need for further research and the development of evidence-based guidelines in managing caustic injury with extensive necrosis in the gastrointestinal tract. Our experience with this rare case highlights the importance of such guidelines in improving patient outcomes.

## Highlights

• Ingestion of caustic substances is a potentially fatal event with high mortality and morbidity rates.
• Unlike children, the ingestion of caustic substances in adults is usually intentional and in larger volumes, which leads to more severe damage.
• Depending on the patient's condition and the injury's severity, management can vary from early discharge to performing severe surgical procedures.
• Developing strictures remains a significant challenge in managing these patients in the late phase.

## 1 Introduction

Ingestion of caustic substances is a potentially fatal event that imposes a significant burden on healthcare systems [1]. Caustic substances cause tissue damage following direct physical contact, such as swallowing. These substances are commonly found in drain cleaners, bleach, detergents, and pool or toilet cleaners [2]. According to the American Association of Poison Control Centers (AAPCC) 2021 annual report, 94,279 exposures were recorded in children five years and younger and 76,832 in adults [3]. In most cases, the ingestion of caustic substances by children occurs as exploratory ingestion. In contrast, adults usually consume larger volumes with the intention of suicide [4]. The damage caused by swallowing corrosive substances consists of two phases, acute and chronic; during the acute phase, tissue damage and perforation are considered, while in the chronic phase, strictures and disorders related to swallowing are considered [5]. Depending on the patient's condition and the injury's severity, decisions about patients can be different from early discharge after initial procedures to hospitalization in the Intensive Care Unit (ICU) and performing surgical procedures [2]. The low prevalence of caustic ingestions has led to a lack of experience among clinicians and evidence-based guidelines for managing these cases [1]. In this report, in line with the SCARE criteria [6], we present a case of caustic ingestion in a patient admitted to our hospital who underwent extensive visceral resection and esophageal reconstruction.

## 2 Presentation of case

A 32-year-old man was brought to the emergency department of our hospital due to swallowing drain cleaner. The patient mentioned drinking a glass of drain cleaner about 8 h ago, and he vomited part of it. There was no exact information on the nature of the ingested drain cleaner, but it was probably alkaline. Past medical and surgical histories were nonspecific. In the initial evaluation, the patient was conscious, and the airway was intact, but he had difficult articulation and hoarseness. Evidence of respiratory distress and the use of secondary respiratory muscles were also detected. There was no evidence of bleeding. He had a blood pressure of 120/80 mmHg, a pulse rate of 114/min, and a SpO_2_ of 95 %. Stridor was auscultated on the respiratory exam. Heart auscultation was normal. On body examination, swelling and burn marks were observed around the mouth, as well as superficial burn marks in the left lower limb. Abdominal examination revealed a board-like abdomen and generalized tenderness.

**Fig. 1:**
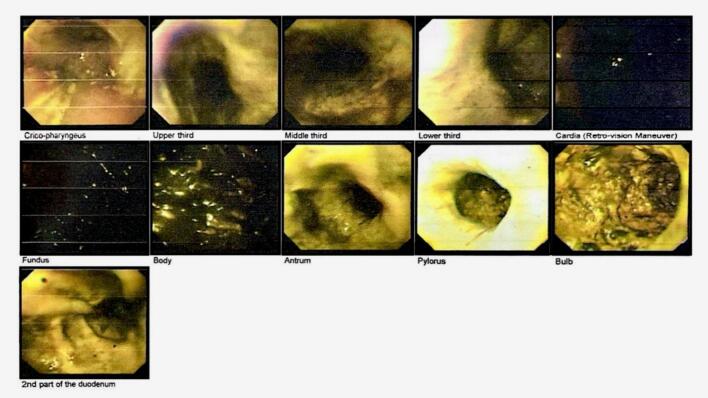
Endoscopic study showing extensive damage to esophagus, stomach, and duodenum.

**Fig. 2:**
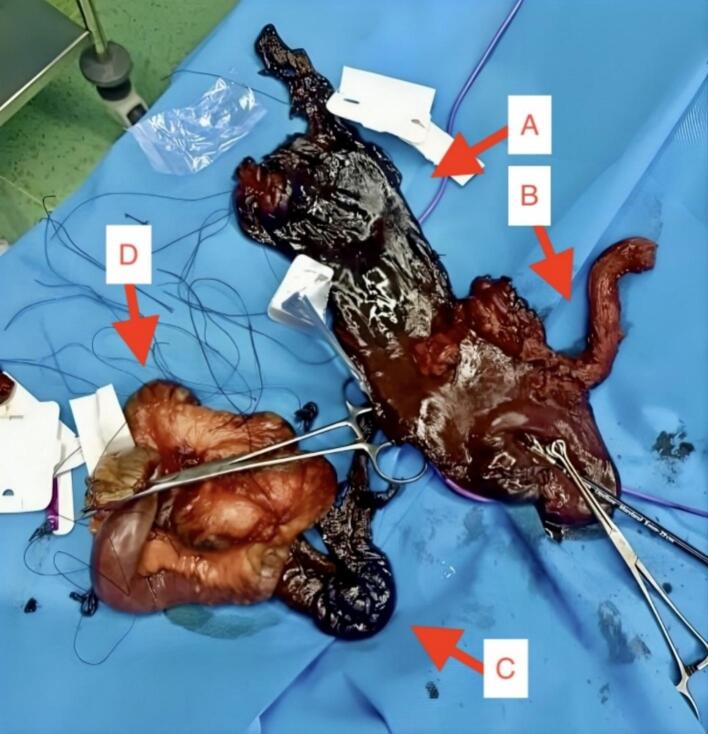
Intraoperative image of resected specimen. A: Stomach; B: Esophagus; C: Duodenum and head of pancreas; D: Jejunum.

In laboratory blood tests, Cell Blood Count (CBC) indicated a Leukocytosis (25.1 × 103/mm3). In Venous Blood Gas (VBG) analysis, a metabolic acidosis (pH 7.13, HCO_3_ 12.6 meq/L, and pCO_2_ 39.4 mmHg) was noted, corresponding to severe tissue damage. There were no other remarkable findings in the laboratory study. An intravenous line and a Foley catheter were installed for the patient, and he was resuscitated with crystalloid (Normal Saline 1 Liter) and prescribed antibiotics intravenously (Ceftriaxone 2 g per day, and Metronidazole 1.5 g per day). Then, the patient underwent endoscopy, and according to Zargar's scoring system, grade 2A-2B damage in the esophagus, 2B-3A in the stomach, and 2A-2B in the duodenum were shown (Fig. 1). Due to extensive damage and clinical evidence of peritonitis, the patient was transferred to the operating room for further intervention, in which exploratory laparotomy was done after intubation and induction of general anesthesia. The patient's abdomen was explored by a midline incision, and evidence of transmural necrosis was seen in the stomach, duodenum, jejunum up to 20 cm after the ligament of Treitz and the pancreas head. After releasing the lesser and greater curvatures, gastrectomy was performed. Then, the patient's esophagus was released by cervical incision, and Orringer esophagectomy was done. In addition, because of extensive necrosis in the duodenum, jejunum, and head of the pancreas, Whipple's procedure was performed, during which the jejunum was anastomosed to the hepatic duct and pancreas by invagination technique. Finally, a jejunostomy tube was inserted to feed the patient post-operation. The specimens removed intraoperatively were sent for pathological examination, which was consistent with the intraoperative findings and indicated transmural necrosis of the esophagus, stomach, and small intestine, along with areas of necrosis in the pancreas head parenchyma (Fig. 2). After surgery, the patient was transferred to the ICU and underwent correction of electrolyte levels and pH disturbances. He was given Heparin (5000 units three times a day) as deep vein thrombosis (DVT) prophylaxis. In the acute phase of management and also during his ICU residence, he did not need a blood transfusion.

Observing the biliary green discharge in the patient's drain within 48 h after surgery suspected leakage. Considering the clinical conditions of the patient who had recently undergone a major surgery and the very high risk of another surgery, a non-surgical plan was preferred, in which the patient underwent Total Parenteral Nutrition (TPN). Ten days later, while the evidence of leakage disappeared, enteral feeding was started through the jejunostomy tube under the supervision of a nutritionist, and after a few days, he started defecating. After two weeks, the patient was transferred to the surgery ward. He was out of bed and started mobilization gradually with the help of the nursing team, using an abdominal binder. Finally, after five days of hospitalization in the surgery ward, the patient was discharged. Six months later, the patient underwent Colon Interposition, in which the transverse colon and a part of the descending colon were placed in the retrosternal region and anastomosed to the remaining part of the esophagus. After one year, because of developing stricture in the anastomosis site, despite several dilation procedures, the patient was unable to be fed orally. Therefore, he underwent enteral feeding and Partial Parenteral Nutrition (PPN) to provide adequate calories. Eventually, strictureplasty was done through cervical incision.

## 3 Discussion

Unlike children, the ingestion of caustic substances in adults is usually done intentionally and in larger volumes. As a result, these cases usually cause more severe damage [4]. In intentional cases, alkalis are more commonly swallowed than acids [7], and unlike acids, by causing liquefaction necrosis and saponification of fats, dissolve the tissue and create a deeper penetration [8]. The damage caused by swallowing corrosive substances includes acute and chronic phases. Manifestations in the acute phase vary widely and include mouth, substernal, or epigastric area pain, swelling of the mouth and tongue, dysphagia and odynophagia, hypersalivation, and vomiting. In addition, with less prevalence, damage to the larynx and epiglottis can manifest as stridor, change in voice, and difficulty breathing. In more severe injuries following perforation of the esophagus and stomach, there may be evidence of mediastinitis and peritonitis. The presence of fever, tachycardia, and shock usually indicate the existence of a more extensive and severe injury [1,2,9]. The most common complication of patients in the late phase and after a few weeks to a few months is stricture, which manifests in complaints of chronic pain and the reappearance of dysphagia [2,10]. Some patients will develop esophageal carcinoma over decades [11]. The management of patients with caustic injuries begins with securing the airway, fluid resuscitation, and pain control. Laryngoscopy should be performed to evaluate the airway and determine the need for intubation in patients with respiratory distress [1]. Examination of the whole body, especially the face, should be done, even though normal examination can't rule out gastroesophageal damage, so early esophagoscopy is indicated in the first 48 h and preferably in the first 24 h, as we did [1,12]. According to endoscopic findings, the amount of injury is evaluated based on the grading system stated by Zargar et al. [13]. Standard laboratory tests have been recommended in critical patients, which can be normal initially. Still, leukocytosis, high C-reactive protein (CRP), severe acidosis, renal failure, impaired Liver Function Test (LFT), and thrombocytopenia are findings in favor of transmural necrosis. They are associated with worse outcomes as found in our patient [1]. Broad-spectrum antibiotics, Proton-Pump Inhibitors (PPIs), and steroids are routinely used; however, there is no evidence of their benefits in all patients, and we didn't use them to manage our case. Emetics and nasogastric tube insertion are not useful in caustic injury management. Also, the use of neutralizers of swallowed substances is not recommended due to causing heat damage and increasing the risk of vomiting [14,15]. Patients with low-grade injuries (grade 1-2A) do not need surgery and can be discharged after supportive care [16]. On the other hand, evidence of transmural necrosis or perforation is the most important indication for surgery [17]. In a study of 348 caustic ingestion cases in Iran, the majority of patients were men 18 to 35 years old. This study showed that 15.2 % of cases needed ICU admission, 33 % needed surgical interventions, and 8 % died. Factors that are associated with a higher probability of needing surgery include suicidal intent, higher grade of mucosal damage, high volume of the swallowed substance, low level of consciousness at the time of admission, acid-base balance disturbances, and dyspnea, all of which were present in our case [7]. Surgical procedures are specific to each patient, and esophagogastrectomy through a combined abdominal and cervical approach is the most common surgical method in cases of severe gastrointestinal injuries. In 20 % of cases, removing more abdominal organs such as the spleen, colon, small intestine, duodenum, and pancreas is needed [17]. Pancreatoduodenectomy is performed in a small number of patients with duodenal necrosis, as in our case, after which immediate pancreaticobiliary reconstruction is recommended if possible. Pancreatoduodenectomy following caustic injury has a high mortality (up to 50 %) and morbidity (up to 100 %) [18,19]. A jejunostomy tube can be inserted during the first surgery to restore the patient's enteral feeding [14]. In patients who have undergone esophagectomy or in patients with strictures that have not responded to dilatation treatment, esophageal reconstruction is indicated, and the most common procedure is retrosternal colon interposition [20]. Studies have shown that a delay of at least six months in reconstruction due to wound stabilization has been associated with a lower rate of anastomosis-related strictures and better performance [21]. So far, no randomized clinical trial has been conducted to determine which colon is preferable for this operation. However, evidence shows similar results when using either the right or left colon. The mortality rate of colon transposition has been reported from 0 to 10 %, and its morbidity from 19 to 63 %. Considering the importance of patient cooperation in the reconstruction process, as well as the possibility of re-attempting suicide after this operation, it is vital to control the underlying psychiatric disorders [1,22]. The prognosis of patients varies depending on the extent of esophageal damage and underlying medical conditions [13]. Due to the increased risk of developing esophageal carcinoma following caustic injury, it is recommended to start esophageal cancer screening 10–20 years after exposure and repeat it every 2–3 years [23].

## 4 Conclusion

Considering the low prevalence of caustic ingestions and lack of experience among clinicians, we decided to present a rare case of caustic ingestion with massive gastrointestinal damage. To the best of our knowledge, no similar case involving such extensive gastrointestinal damage resulting from caustic ingestion has been published to date. Due to potentially life-threatening conditions in these cases, like in other traumatic patients, management should start by stabilizing the patients. One of the most critical aspects in managing these patients is determining the necessity of surgery by evaluating the findings of physical examinations and early endoscopic studies, which can be life-saving in some cases like this one. Long-term follow-up of these patients is of great importance because of the high risk of esophageal carcinoma and stricture. Developing strictures remains a significant challenge in managing these patients, especially considering the poor nutritional status of these patients and the existence of limitations in performing multiple surgeries.

## Ethical approval

Ethical approval for this study was provided by the Ethical Committee of Madani Hospital, Karaj, Iran. This study complies with ethical and research standards involving humans.

## Funding

No funding was received.

## Author contribution

All authors have read and approved the final manuscript. RB: scientific supervision and performing surgical procedures. AS, MP, MSBC: writing the paper and data collection. JZB: performing the surgical procedures. ZI: reviewing the manuscripts.

## Guarantor

Ahmadreza Sadeghi.

## Consent

Written informed consent was obtained from the patient for publication and any accompanying images. A copy of the written consent is available for review by the Editor-in-Chief of this journal on request.

## Conflict of interest statement

None.
